# Factors associated with drug checking service utilization among people who use drugs in a Canadian setting

**DOI:** 10.1186/s12954-020-00454-4

**Published:** 2020-12-14

**Authors:** Viseth Long, Jaime Arredondo, Lianping Ti, Cameron Grant, Kora DeBeck, M-J Milloy, Mark Lysyshyn, Evan Wood, Thomas Kerr, Kanna Hayashi

**Affiliations:** 1 British Columbia Centre on Substance Use, 400-1045 Howe Street, Vancouver, BC Canada; 2 Faculty of Health Sciences, Simon Fraser University, 8888 University Drive, Burnaby, BC Canada; 3 Department of Medicine, Faculty of Medicine, University of British Columbia, Vancouver, BC Canada; 4 School of Public Policy, Simon Fraser University, Vancouver, BC Canada; 5 Vancouver Coastal Health Authority, Vancouver, BC Canada; 6 School of Population and Public Health, University of British Columbia, Vancouver, BC Canada

**Keywords:** Fentanyl, Drug checking, Drug dealing, Opioids, Overdose, Vancouver

## Abstract

**Background:**

The United States and Canada are amidst an opioid overdose crisis, with the Canadian province of British Columbia (BC) among the hardest hit. In response, drug checking services (DCS) have been introduced in this setting as a novel pilot harm reduction intervention though little is known about usage rates. Therefore, we sought to identify factors associated with drug checking uptake among people who use drugs (PWUD) in Vancouver, BC.

**Methods:**

Data were derived from three ongoing prospective cohort studies of PWUD in Vancouver between June and November 2018. Multivariable logistic regression was used to determine factors associated with self-reported DCS utilization in the past 6 months among participants at high risk of fentanyl exposure (i.e., those self-reporting illicit opioid use or testing positive for fentanyl via urine drug screen).

**Results:**

Among 828 eligible participants, including 451 (55%) males, 176 (21%) reported recent use of DCS. In multivariable analyses, factors significantly associated with DCS utilization included: homelessness (Adjusted Odds Ratio [AOR] 1.47; 95% Confidence Interval [CI] 1.01–2.13) and involvement in drug dealing (AOR 1.59; 95% CI 1.05–2.39).

**Conclusions:**

In our sample of PWUD, uptake of DCS was low, although those who were homeless, a sub-population known to be at a heightened risk of overdose, were more likely to use the services. Those involved in drug dealing were also more likely to use the services, which may imply potential for improving drug market safety. Further evaluation of drug checking is warranted.

## Introduction

An opioid overdose crisis continues to impact communities across the United States and Canada. This is attributed in large part to the introduction of potent and illegally manufactured synthetic opioids into the illicit drug supply [1–4]. In 2018, there were 21.7 opioid overdose deaths per 100,000 population in the United States (US) and 12.4 deaths per 100,000 population in Canada [5, 6]. In the Canadian province of British Columbia (BC), one of the hardest hit regions, a public health emergency was declared in 2016 in response to the increase in illegal drug overdose deaths [7, 8]. As part of the response, various interventions were implemented and scaled up, including widespread availability of naloxone and supervised consumption services. In 2019, the number of overdose deaths has finally declined compared to previous years; however, the rate of death from overdose remains unacceptably high [8]. In 2019, a fatal opioid overdose rate was 19.4 deaths per 100,000 individuals in BC [8].

In this context, drug checking gained attention as a harm reduction intervention with potential to mitigate overdose risks. Drug checking services (DCS) seek to provide people who use drugs (PWUD) with personalized information on the chemical composition of their drugs and increase awareness of their exposure to harmful adulterants [9–12]. Historically, DCS have been implemented mostly in party and music festival settings around the world but have not been well deployed in other drug scenes [11–15]. Amidst an opioid overdose crisis, drug checking creates an opportunity to provide harm reduction education, counselling, and referrals to other services (e.g., drug treatment), as well as monitor trends in the illicit drug supply market [9, 10]. In BC, DCS outside of party and music festival settings was introduced at Insite, a supervised injection facility (SIF) in Vancouver, in 2016 [7]. As described elsewhere [16], the drug-checking technologies used in this setting included fentanyl immunoassay strips in 2016 and the combination of Fourier-Transform Infrared (FTIR) Spectroscopy and fentanyl immunoassay strips since 2017. The FTIR is capable of identifying and quantifying a wide range of substances in mixture submitted for analysis, while the testing strips can qualitatively detect a low concentration of fentanyl presence in the drug sample [7, 11].

Despite the enthusiasm about drug checking, there is limited scientific evaluation of drug checking as a harm reduction intervention, specifically in the context of regular non-medical opioid use and outside of party and music festival settings. Available evidence paints a mixed picture. Emerging research from the US has suggested high levels of willingness to use drug checking among PWUD amidst the opioid overdose crisis and indicated that DCS may encourage them to adopt available harm reduction practices through increased awareness of adulterants, such as fentanyl [9, 10]. For example, a 2017 study involving 334 street-based PWUD found that 85% of participants were interested in using drug checking. Of those interested, 70% expressed that they would change their drug-using behaviors (e.g., discard drugs or dose reduction) if they knew their drug had fentanyl [10]. This was in line with findings of studies focusing on party and festival settings suggesting that many people whose drug samples tested positive for adulterants such as fentanyl discarded their substances [13, 14, 17]. However, a study conducted at Insite in Vancouver found low utilization of a pilot drug checking service using the test strips only, with only approximately 1% of visits to Insite resulting in the use of drug checking [18]. Among those samples tested positive for fentanyl pre-consumption, only 11% planned to discard their drugs [18]. Findings from a qualitative study shed some light on possible reasons for the low uptake of DCS among this population, including time dedication (i.e., time to get drugs checked), a hesitancy to give up drugs, and the accessibility of the services (i.e., limited hours and locations of operation) [19].

Taken together, the literature suggests that there could be some crucial differences in the uptake and effect of drug checking across different drug-using populations and different settings. Therefore, this study sought to identify the prevalence of and factors associated with DCS use among PWUD at a high risk of fentanyl exposure in Vancouver.

## Methods

### Study procedures

Data for this study was derived from three ongoing prospective cohort studies of people who use drugs (PWUD) from Vancouver, Canada: the Vancouver Injection Drug Users Study (VIDUS), the AIDS Care Cohort to evaluate Exposure to Survival Services (ACCESS), and the At-Risk Youth Study (ARYS). Detailed sampling and recruitment procedures for these cohorts have been described elsewhere [20–22]. In brief, VIDUS enrolls HIV-negative adults (≥ 18 years of age) who injected drugs in the month prior to enrolment; ACCESS enrolls HIV-positive adults who used illicit drugs other than or in addition to cannabis in the month before enrolment, and ARYS enrolls street-involved youth aged 14–26 years who used illicit drugs other than or in addition to cannabis in the month before enrolment. In all studies, the primary modes of recruitment were self-referral, word of mouth, and street outreach. Eligibility criteria included residing in the Greater Vancouver region and providing written informed consent. The study instruments and follow-up procedures, including the questionnaires, were harmonized to permit for pooled data analyses. At baseline and semi-annually thereafter, participants completed an interviewer-administered questionnaire that elicited information about socio-demographic characteristics, drug use and other behavioral patterns and social/structural exposures. Additionally, participants completed a urine drug screen (UDS) using BTNX Rapid Response™ Multi-Drug Test Panel (Markham, ON, Canada). The procedure for UDS has been described elsewhere [23]. At the completion of each study visit, participants were provided with a $40 (CDN) honorarium. The cohort studies have received approvals from the University of British Columbia/Providence Health Care Research Ethics Board.

### Study sample and primary outcome measure

The present analysis is a cross-sectional study embedded within the prospective cohort studies. The sample was restricted to individuals who completed a study visit between June and November 2018, as questions related to DCS were added to the questionnaire during this period. The sample was further restricted to those who self-reported any illicit opioid use (i.e., heroin, non-medical use of prescription opioids, or suspected use of fentanyl) in the past 6 months or tested positive for fentanyl through the UDS at the time of the interview, and those who provided a valid answer to the question about the primary outcome. The primary outcome of interest was self-reported DCS utilization in the past 6 months (yes vs. no) and derived from the question: “Have you used any drug checking services in the last 6 months?” If responded “no”, participants were asked why they didn’t use drug checking services and provided with a list of options including, “Service wasn’t easily accessible to me (location, times, etc.)”, “Not interested in knowing what’s in my drugs”, “No point in getting my drugs checked, as I have no other alternates (e.g., there’s fentanyl in everything, dope sick, desperate)”, “Other (i.e., an open-ended option)”, among other responses.

### Study variables

We considered a range of explanatory variables, including demographic, behavioral, and other socio-structural characteristics, that might be associated with DCS utilization, based on known correlates of accessing other harm reduction services among this population [2, 24–27]. Socio-demographic data included: self-identified gender (male vs. non-male); age (per year older); homelessness (yes vs. no); ethnicity/ancestry (white vs. non-white); and living in the Downtown Eastside neighborhood (DTES) of Vancouver that is an epicenter of illicit drug use (yes vs. no). Drug use behavior variables were dichotomous (yes vs. no) and included: injection drug use; ≥ daily heroin use; ≥ daily non-medical use of prescription opioids; ≥ daily crystal methamphetamine use; ≥ daily benzodiazepine use; ≥ daily crack cocaine use; ≥ daily cocaine use; binge drug use; and non-fatal drug overdose. Other social and structural exposures included: incarceration (yes vs. no); and involvement in drug dealing (yes vs. no). All behavioral and social/structural variables were based on self-report and referred to the previous 6 months unless otherwise specified.

### Statistical analysis

First, we used logistic regression to examine bivariable associations between the explanatory variables and the primary outcome variable. Then, we used an a priori- defined backward model selection procedure based on examination of Akaike Information Criterion (AIC) to fit a multivariable model [28]. In brief, we first included a full multivariable model with all explanatory variables that were significantly associated with the drug checking services utilization at *p* < 0.10 in the bivariable analyses, excluding injection drug use in the past 6 months due to the skewed distribution of responses (i.e., virtually all participants [94.9%] who used drug checking injected drugs in the past 6 months). After examining the AIC value of the model, we removed the variable with the largest *p* value and built a reduced model. We continued this iterative process and selected the multivariable model with the lowest AIC score. In a sub-analysis, we used descriptive statistics to examine self-reported reasons for not having used drug checking services. All *p* values were two-sided. All statistical analyses were performed using SAS, version 9.4 (SAS Institute Inc., Cary, NC, USA).

## Results

A total of 828 individuals were eligible for the present study, of whom 451 (55%) reported being male, and 390 (47%) being white. The median age of this sample was 41.0 (quartile Q1–Q3 = 30.6–52.1) years. Of this sample, 176 (21%) reported DCS use in the past 6 months, of whom 93 (53%) were male, and almost all (167, 94.9%) reported injection drug use in the past 6 months. Characteristics of the sample and the results of the bivariable logistic regression analyses are presented in Table 1.

**Table 1:** Bivariable analyses of factors associated with recent use of DCS among PWUD in Vancouver (*n* = 828)

Characteristic	Drug checking service use		Odds ratio (95% CI)	*p* value
	Yes *n* (%) *n* = 176 (21.3%)	No *n* (%) *n* = 652 (78.7%)		
Age (med, Q1–Q3)	39.5 (30.0–51.6)	41.3 (30.8–52.4)	1.00 (0.98–1.01)	0.577
Gender				
Male	93 (52.8)	358 (54.9)	1.03 (0.71–1.49)	0.896
Non-male	56 (31.8)	221 (33.9)		
Ethnicity				
White	90 (51.1)	300 (46.0)	1.25 (0.89–1.75)	0.192
Non-White	83 (47.2)	346 (53.1)		
Living in DTES*				
Yes	131 (74.4)	402 (61.7)	1.81 (1.25–2.65)	0.002
No	45 (25.6)	250 (38.3)		
Drug dealing*				
Yes	49 (27.8)	111 (17.0)	1.87 (1.26–2.75)	0.002
No	127 (72.2)	539 (82.7)		
Homelessness*				
Yes	62 (35.2)	158 (24.2)	1.71 (1.19–2.44)	0.003
No	113 (64.2)	493 (75.6)		
Incarceration*				
Yes	19 (10.8)	44 (6.7)	1.67 (0.93–2.90)	0.076
No	157 (89.2)	607 (93.1)		
Non-fatal Overdose*				
Yes	38 (21.6)	106 (16.3)	1.42 (0.93–2.13)	0.101
No	138 (78.4)	545 (83.6)		
Binge drug use*^†^				
Yes	75 (42.6)	241 (37.0)	1.26 (0.90–1.77)	0.176
No	101 (57.4)	410 (62.9)		
Injection drug use*				
Yes	167 (94.9)	522 (80.1)	4.62 (2.43–9.97)	< 0.001
No	9 (5.1)	130 (19.9)		
Daily Benzodiazepine use*^†^				
Yes	3 (1.7)	4 (0.6)	2.81 (0.55–12.87)	0.179
No	172 (97.7)	645 (98.9)		
≥ Daily crack use*^†^				
Yes	9 (5.1)	76 (11.7)	0.41 (0.19–0.79)	0.014
No	167 (94.9)	576 (88.3)		
≥ Daily cocaine use*^†^				
Yes	2 (1.1)	25 (3.8)	0.29 (0.05–0.98)	0.093
No	174 (98.9)	627 (96.2)		
≥ Daily heroin Use*^†^				
Yes	89 (50.6)	260 (39.9)	1.54 (1.10–2.16)	0.011
No	87 (49.4)	392 (60.1)		
≥ Daily methamphetamine use*^†^				
Yes	53 (30.1)	146 (22.4)	1.49 (1.03–2.16)	0.034
No	123 (69.9)	506 (77.6)		
≥ Daily non-medical use of prescription opioids*^†^				
Yes	10 (5.7)	16 (2.5)	2.39 (1.03–5.30)	0.034
No	166 (94.3)	636 (97.5)		

CI, confidence interval; DTES, downtown eastside; Q, quartile; med, median

^*^Denotes activities/events in the past 6 months

^†^Refers to any route of consumption (i.e., sniffing, snorting, smoking or injecting)

Table 2 shows the results of the multivariable logistic regression analyses. Factors independently associated with DCS use included: homelessness (Adjusted Odds Ratio [AOR] 1.47; 95% confidence interval [CI] 1.01–2.13), living in the DTES (AOR 1.68; 95% CI 1.14–2.47), recent involvement in drug dealing (AOR 1.59; 95% CI 1.05–2.39) and at least daily crack use (AOR 0.40; 95% CI 0.19–0.83).

**Table 2:** Multivariable logistic regression analyses of factors associated with recent use of DCS among PWUD (*n* = 828)

Variable	Adjusted odds ratio (AOR)	95% Confidence interval (CI)	*p* value
Living in DTES*			
(Yes vs. No)	1.68	(1.14–2.47)	0.009
Drug dealing*			
(Yes vs. No)	1.59	(1.05–2.39)	0.027
Homelessness*			
(Yes vs. No)	1.47	(1.01–2.13)	0.042
≥ Daily cocaine use*†			
(Yes vs. No)	0.26	(0.06–1.15)	0.075
≥ Daily crack use*†			
(Yes vs. No)	0.40	(0.19–0.83)	0.014
≥ Daily heroin use*†			
(Yes vs. No)	1.35	(0.95–1.91)	0.095
≥ Daily non-medical use of prescription opioids*†			
(Yes vs. No)	2.35	(0.98–5.60)	0.055

DTES, downtown eastside

^*^Denotes activities/events in the past 6 months

^†^Refers to any route of consumption (i.e., sniffing, snorting, smoking or injecting)

As shown in Table 3, commonly reported reasons for not using DCS included “not interested in knowing the drug contents” (33%), “no alternatives (fentanyl in everything, dope sick, desperate)” (24%), “unaware of service” (24%), and “service wasn’t easily accessible (location, times, etc.)” (13%).

**Table 3:** Self-reported reasons for not using drug checking services among PWUD in Vancouver (*n* = 665)

Reasons	*N*	%
Not interested in knowing the drug contents*	220	33.1
No alternatives (fentanyl in everything, dope sick, desperate)	159	23.9
Unaware of service	157	23.6
Service wasn’t easily accessible (location, times, etc.)	83	12.5
Use known source/dealer	17	2.6
Rarely use drugs	10	1.5
Use stimulants	3	0.5
Plan to use drug checking	3	0.5
Other	13	2.0

^*^Refers to any type of drug

Participants could provide more than one response

## Discussion

In our sample of PWUD at a high risk of fentanyl exposure in Vancouver, we found that the uptake of DCS was low, with only one-in-five using the services in the past 6 months. In multivariable analysis, those who lived in DTES, were homeless or were involved in drug dealing were more likely to use DCS, while those who used crack cocaine at least daily were less likely to use DCS. The most commonly reported reason for not using DCS was no interest in knowing the contents of the drug.

The low uptake of DCS identified by our study is different from a finding of a previous study in the US showing a high willingness to use DCS among street-based samples of PWUD [10]. This confirms that willingness to use DCS and the actual uptake of DCS are two very different measures. Our results of the sub-analysis suggest that low uptake of DCS may reflect both the high levels of penetration of fentanyl into the illicit drug market in our setting [16] as well as the limited awareness and availability of this service during the study period. As a result of the current drug market and limited financial resources, people’s ability to obtain new drugs once their drugs test positive for adulterants is limited. Thus, people may not place a high value on DCS. However, we note that there are other harm reduction strategies (e.g., dose reduction, not using alone, using at an SCS or OPS) that could be used once DCS confirm the presence of fentanyl in a drug sample. In this regard, the use of FTIR may have some added value in fentanyl saturated markets because it can provide PWUD with the percentage of fentanyl in their drug samples. Future research should investigate awareness and uptake of these other harm reduction strategies after the use of DCS. On the other hand, among those who did not use DCS in our sample, nearly one-fifth reported that they were unaware of the service, and some reported programmatic barriers to access the service (e.g., location, opening hours). Of note, during a large part of this study period, DCS were only operating at a single SIF in Vancouver with limited hours. It is also worth mentioning that there were more DCS available in the area after the study period. Although DCS is located mostly in SIFs and harm reduction centers people can come in and check their drugs without using SIF services as well. Therefore, ongoing monitoring of DCS uptake is needed as DCS sites expand.

Even though we observed the limited reach of DCS to PWUD in our setting, we also found that those who were homeless were more likely to use the service. This finding suggests that the service in our setting have reached a sub-population at a particularly high risk of overdose [29–31]. This likely reflects that services have been provided in a low-threshold manner as it was housed in a SIF designed to serve socially marginalized populations [32]. Nevertheless, because the majority (72%) of fatal overdoses were occurring inside private residences between 2016 and 2017 [33], future research should investigate how DCS can reach PWUD who use drugs alone at home as well.

Our study revealed a positive association between drug dealing and DCS use. This is consistent with previous research showing that people who had recently been involved in drug dealing were more likely to be willing to use DCS [34]. Drug dealers can play an essential role in reducing drug-related harms for PWUD through information sharing with their clients regarding the content of their drugs. Previous qualitative research reported that PWUD were less likely to use DCS when they had an established relationship with drug dealers [24]. This comes with an underlying assumption that drug dealers know the content of the drugs they are selling, and their willingness to share the information with their clients. However, due to the inconsistent nature of the drug market in our setting, it is unlikely that street-level dealers accurately know the content of their drugs. Moreover, participants in our study are more likely to be street-level dealers. To make big impact, we would need to get at the supplier or distribution level dealers. Nevertheless, the ability for street-level dealers to utilize DCS to check their drugs before selling still has potential indirect benefits to PWUD as a harm reduction measure to prevent drug-related harms (e.g., overdose). Further engagement and research with different levels of drug dealers in drug checking may be warranted to improve the DCS design, delivery and effectiveness.

Finally, we found that those who frequently used crack cocaine were less likely to use DCS. These findings suggest a possible association between the preferred substance type (opioids vs. stimulants) and the uptake of DCS. Results from the British Columbia Centre on Substance Use’s provincial drug checking reports [35–40] show that the proportion of stimulants submitted for drug checking was low compared to opioids. Of all the stimulant samples tested, less than one percent tested positive for fentanyl compared to 88% for opioid samples [35–40]. The low uptake of DCS among frequent stimulant users might reflect a perceived low risk of fentanyl exposure in stimulants compared to opioids. However, when a stimulant drug is mixed with fentanyl, opioid-naïve stimulant users are at a heightened risk of overdose due to low tolerance [41] as well as the lack of awareness of fentanyl presence in their drugs. As such, providing accurate information to stimulant users regarding the contents of their drugs via DCS could play a critical role in reducing overdose risk. Therefore, expanding the DCS to non-opioid users may be required [41, 42].

### Limitations

There are several limitations to this study. First, data from our study were collected from non-random samples of PWUD, and therefore may not be generalizable to other populations of PWUD. Second, except for UDS screens, data were collected using self-reported information and thus is subject to reporting biases, including recall bias and socially desirable responding. Finally, it is impossible to determine the temporality between the outcome and explanatory variables from this cross-sectional study.

## Conclusions

In summary, the recent use of DCS was low among our sample of PWUD at a high risk of fentanyl exposure. However, it appears to have reached sub-populations at a heightened risk of overdose, including those who were homeless. Moreover, people who were involved in drug dealing were more likely to use DCS, which may imply the potential for improving drug market safety. Findings also revealed that stimulant users were less likely to use DCS, suggesting further engagement with stimulant users is warranted in future planning for DCS implementation. As DCS continue to evolve, further evaluation of DCS is needed.

## Data Availability

The data used for this study is not publicly available. For further information on the data and materials used in this study, please contact the corresponding author.

## Abbreviations

DCS: Drug checking services
DTES: Downtown eastside
FTIR: Fourier-transform infrared
OPS: Overdose prevention sites
PWUD: People who use drugs
SCS: Supervised consumption sites
SIF: Supervised injection facility
UDS: Urine drug screen
